# Inhalation Toxicity of Humidifier Disinfectants as a Risk Factor of Children’s Interstitial Lung Disease in Korea: A Case-Control Study

**DOI:** 10.1371/journal.pone.0064430

**Published:** 2013-06-05

**Authors:** Hyeon-Jong Yang, Hwa-Jung Kim, Jinho Yu, Eun Lee, Young-Ho Jung, Hyung-Young Kim, Ju-Hee Seo, Geun-Yong Kwon, Ji-Hyuk Park, Jin Gwack, Seung-Ki Youn, Jun-Wook Kwon, Byung-Yool Jun, Kyung Won Kim, Kangmo Ahn, Soo-Young Lee, June-Dong Park, Ji-Won Kwon, Byoung-Ju Kim, Moo-Song Lee, Kyung-Hyun Do, Se-Jin Jang, Bok-Yang Pyun, Soo-Jong Hong

**Affiliations:** 1 Department of Pediatrics, Pediatric Allergy and Respiratory Center, Soonchunhyang University Hospital, Soonchunhyang University College of Medicine, Seoul, Korea; 2 Department of Clinical Epidemiology and Biostatistics, Asan Cancer Center, Asan Medical Center, University of Ulsan College of Medicine, Seoul, Korea; 3 Department of Pediatrics, Childhood Asthma Atopy Center, Asan Medical Center Children’s Hospital, University of Ulsan College of Medicine, Seoul, Korea; 4 Research Center for Standardization of Allergic Diseases, Asan Medical Center Children’s Hospital, University of Ulsan College of Medicine, Seoul, Korea; 5 Department of Pediatrics, Korean Cancer Center Hospital, Seoul, Korea; 6 Division of Epidemic Intelligence Service, Korea Centers for Disease Control and Prevention, Osong, Korea; 7 Center for Infectious Disease Surveillance and Response, Korea Centers for Disease Control and Prevention, Osong, Korea; 8 Department of Pediatrics and Institute of Allergy, Biomolecule Secretion Research Center, Yonsei University College of Medicine, Seoul, Korea; 9 Department of Pediatrics, Samsung Medical Center, Sungkyunkwan University School of Medicine, Seoul, Korea; 10 Department of Pediatrics, Ajou University Hospital, Ajou University School of Medicine, Suwon, Korea; 11 Department of Pediatrics, Seoul National University Children's Hospital, Seoul University College of Medicine, Seoul, Korea; 12 Department of Pediatrics, Seoul National University Bundang Hospital, Seoul University College of Medicine, Seungnam, Korea; 13 Department of Pediatrics, Inje University Haeundae Paik Hospital, University of Inje College of Medicine, Busan, Korea; 14 Department of Preventive Medicine, Asan Cancer Center, Asan Medical Center, University of Ulsan College of Medicine, Seoul, Korea; 15 Department of Radiology, Asan Medical Center, University of Ulsan College of Medicine, Seoul, Korea; 16 Department of Pathology, Asan Medical Center, University of Ulsan College of Medicine, Seoul, Korea; University of Rochester Medical Center, United States of America

## Abstract

**Background:**

The occurrence of numerous cases of interstitial lung disease in children (chILD) every spring in Korea starting in 2006 raised suspicion about a causal relationship with the use of humidifier disinfectants (HDs). The aim of this study was to evaluate the association between HD use and the risk of chILD.

**Methods:**

This retrospective, 1∶3 matched case-control study consisted of 16 cases of chILD that had developed between 2010 and 2011. The three groups of parallel controls (patients with acute lobar pneumonia, asthma, and healthy children) were matched by age, gender, and index date. Indoor/outdoor environmental risk factors, including HD use, were investigated by asking the guardians to complete a questionnaire.

**Results:**

The median age of the affected children (43.8% male) was 26 months (18.25–36.25). The chILD group did not differ significantly from the control groups with respect to socio-demographic and clinical variables. Indoor and outdoor environmental factors were not associated with a risk of chILD. However, the previous use of HDs (OR; 2.73. 95% CI; 1.41–5.90, *P* = 0.00) were independently associated with an increased risk.

**Conclusions:**

This study showed that HDs, which are widely used in South Korea in the winter season, independently increased the risk of chILD in spring. Therefore, continuous monitoring and, if needed, changes in policy are essential to prevent and control pediatric diseases caused by toxic chemicals.

## Introduction

Children’s interstitial lung disease (chILD) refers to a diverse spectrum of rare and diffuse lung pathologies that are associated with high morbidity and mortality. [1] Various causes of chILD including infection, environmental agents, radiation, medications, genetic predisposition, metabolic storage diseases, and autoimmune diseases have been identified. However, a considerable number of ChILD cases are still idiopathic. [2].

Recently, we reported several cases of unclassified interstitial pneumonia with fibrosis, describing it as a new entity of chILD. [3] Briefly, these cases were the young children and parents of two families who coincidentally developed the disease in spring. In each of these cases, the same clinico-radiologic-pathological findings were noted, including a clinically rapid progression to respiratory failure. The histopathological findings showed a centrilobular distribution of alveolar damage with sparing of the peripheral lung parenchyma. While the rapid progression and radiological findings were similar to what is seen in hypersensitivity pneumonitis, the pathological findings and clinical course (poor response to the treatment and a poor prognosis) were quite different. Interestingly, causative microorganisms were not detected, despite the spring-time occurrence of the disease.[3]–[5] Thus, the cause was suspected to be either an inflammatory process interacting with an altered immune response against an appropriate genetic background, or as-yet unidentified environmental factors which the household members were evenly exposed at the same time, particularly in winter. At our institution, a total 51 children were diagnosed between 2006 and 2011 with a similar type of chILD, namely, chILD characterized by rapid progression and a poor prognosis, as indicated by the high mortality (43.13%) (unpublished data). Sporadic epidemics of similar cases in spring have also been repeatedly reported throughout Korea since 2006. [4], [5].

On May 11, 2011, an article entitled “Dozens of pregnant or postpartum women have simultaneously developed interstitial lung disease of unknown cause and died” made headlines in major a newspaper in South Korea. This article and subsequent reports described the high prevalence of this disease in pregnant and postpartum women and their children, and its 100% mortality rate (all cases who did not undergo lung transplantation died). These serial reports alerted the Korea Centers for Disease Control and Prevention (KCDC), which initiated investigations to identify the cause of the disease and ways to prevent it. On August 31, KCDC announced to the media including all major TV, radio networks, and newspapers, that their preliminary epidemiological analyses of acute lung injury with unknown causes in adult cases revealed that while these cases were not associated with microorganisms, a strong association with the use of humidifier disinfectants (HDs) (OR, 47.3) had been observed. [6].

Several gene mutations (such as in the ATP-binding cassette transporter A3 or surfactant protein C) have been reported to be risk factors for chILD. [2] However, although a genetic association cannot be excluded, genetics alone cannot fully explain the pathogenesis of chILD, because family members of different ages are affected at the same time. It seems more likely that chILD is the result of environmental factors to which household members, regardless of their genetic predisposition, are evenly exposed. Because HDs are often used in Korea during the winter, it was suspected that they may play a key role in the subsequent development of chILD during the spring. To date, HDs have never been investigated as a risk factor of chILD. Thus, the aim of this hospital-based, case-control study was to evaluate whether there was indeed an association between the use of HDs and the pathogenesis of chILD.

## Materials and Methods

### Study Design

A case-control study designed to compare the environmental exposures of chILD patients with those of their matched controls was conducted at the Department of Pediatrics, Asan Medical Center (a tertiary medical center in Seoul, Korea). The study protocol was approved by the Institutional Review Boards of Asan Medical Center, University of Ulsan College of Medicine, and written informed consent was obtained from the parents or guardians at enrollment.

### Study Subjects: Case Ascertainment

**Figure 1:**
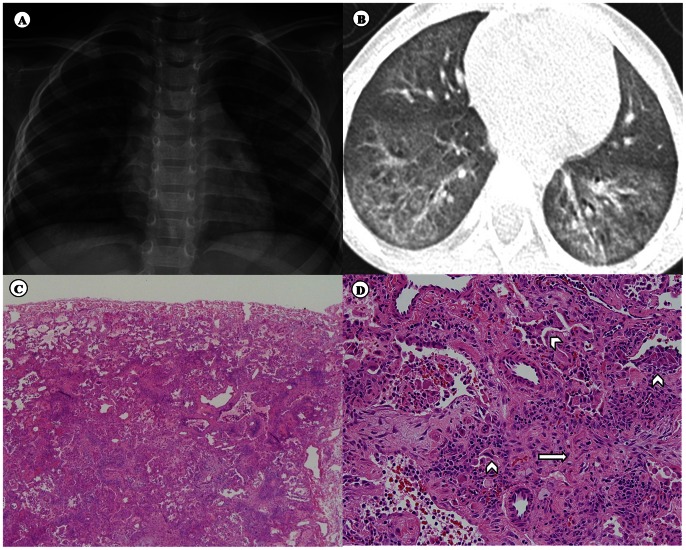
Case ascertainment criteria. (A) Chest radiography showing diffuse, ill-defined ground-glass opacities and several ill-defined small nodular opacities in both lungs (in the 4 weeks after respiratory symptoms started). (B) Chest computed tomography showing diffuse centrilobular nodules in both lungs, with ground-glass opacities suggestive of peribronchiolar fibrosis (in the 4 weeks after respiratory symptoms started). (C) The most striking histological feature, interstitial thickening and fibrosis with a centrilobular distribution and relative sparing of the subpleural parenchyma (upper one-third). Original magnification ×40. (D) Interstitial fibroblasts proliferating in a pale myxoid stroma (arrows) and collapsed alveolar spaces lined by activated pneumocytes (arrowhead). Original magnification ×200.

In total 51 cases were diagnosed with chILD of an unknown cause in our institution between January 1, 2006 and June 30, 2011. Of these, the 16 subjects who were diagnosed between April 1, 2010 and June 30, 2011 were included in the study. These cases were confirmed using clinico-radiologic-pathologic ascertainment criteria (Figure 1). The definite inclusion criteria consisted of all of the following: (1) age less than 18 years at the time of diagnosis; (2) rapid progression to acute respiratory distress; (3) chest tomography findings of ground-glass opacities with a centrilobular distribution, evidence of air leakage in the lung parenchyma, and sparing of the peripheral lung parenchyma; (4) histopathological findings of interstitial thickening and fibrosis with a centrilobular distribution and relative sparing of the subpleural parenchyma; and (5) chILD occurrence in the spring (i.e., from March 1 through to May 31).

The electronic medical records of all patients were reviewed comprehensively, and additional information, including exposure status to environmental factors, was obtained by administering a questionnaire (the interview process and questionnaire contents are detailed in the supplementary text). In addition, to confirm the diagnosis and to better assess the clinical features of each case, the medical records of the patients were reviewed manually by a committee composed of pediatric pulmonologists, radiologists, pathologists, and epidemiologists.

Exclusion criteria were as follows: (1) other types of ILD, such as those due to lung growth abnormalities, storage diseases, or surfactant dysfunction, as identified by histopathological findings; (2) patients with underlying conditions that could be associated with ILD (e.g., chromosomal defects, collagen-vascular diseases, congenital heart diseases, congenital metabolic disorders, congenital lung diseases, immunodeficiency, malignancy, and post-transplantation) and that were present before the index date; (3) ILD in which the cause was established after the index date based on a review of the individual's medical records; or (4) the absence of written informed consent.

### Selection of the Controls

In this 1∶3 matched case-control study, each chILD patient was matched with three controls randomly selected from the following entities: acute lobar pneumonia, which is representative acute respiratory disease; asthma, which is representative chronic respiratory disease; and healthy subjects. All controls were paired with their matched chILD patient by age (within 6 months), gender, and index date. The chILD index date was defined as the date of the initial diagnosis of the disease (within one month). The informed consent was requested for all controls at the time of enrollment. The exclusion criteria were the same as described above for the chILD cases.

### HD Exposure Ascertainment

To determine the exposure status to HDs prior to the index date, a questionnaire that included the questions “Did you ever use a humidifier in the 1 year before the index date?” and “Did you ever use a humidifier disinfectant in the 1 year before the index date?” was developed. To reduce recall bias, the color-printed questionnaire included pictures of common on−/off-market HDs (see supplementary material).

### Other Variables

Data on pertinent variables that could act as covariates or confounders were also collected as follows: (1) personal characteristics, including ethnicity, birth mode, personal history in the 12 months prior to the index date (e.g., passive smoking, overseas trip, medications including oriental medicine, health supplements, pet ownership); (2) familial characteristics, including family history of ILD in the 6 months prior to or after the index date, chronic respiratory/allergic diseases, and socioeconomic status (educational levels of parents); (3) comorbid conditions in the 6 months prior to the index date, including chronic respiratory diseases, chronic liver disease, diabetes mellitus, neurological disease with immobility, chronic renal insufficiency, and autoimmune diseases; (4) factors altering the immune response, including immunosuppressive therapy, general anesthesia, major surgery, and transfusion in the 6 months prior to the index date; (5) indoor environmental factors, including housing, storey of residence, type of heating system, type of drinking water, recent history of house renovation or new furniture, use of air cleanser, air conditioner, air freshener, water purifier, or hair spray in the 12 months prior to the index date; and (6) outdoor environmental factors, including region of residence and presence of particular outside facilities within 2 km (e.g., garbage incinerator, sewage treatment plants, factory, chemical treatment area, plants, farm/orchard, cattle shed/pigsty, or power station). Information from the illustrated questionnaire, which also contained photographs of on−/off-market air cleansers, air conditioners, water purifiers, humidifiers, air fresheners, and hair sprays used in Korea as well as the above-described HDs, was also collected.

### Statistical Analysis

The primary goal of the data analysis was to determine the odds ratio (OR) of HD exposure status for cases and controls. Nominal variables were analyzed by using a *chi-square* or *Fisher’s exact test*. Univariate analyses using conditional logistic regression analysis were conducted to identify all pertinent covariates and confounders, and to assess the comparability of the cases and controls. Depending upon the results of these univariate analyses, we determined factors that need to be adjusted for in all analyses. The OR for the association of chILD with HD exposure was calculated with a 95% confidence interval. All calculated *P* values were two-sided and *P*<0.05 was considered to be statistically significant. The analyses were performed by using SAS software package (SAS Institute, Cary, NC).

## Results

One of the controls, a patient with acute lobar pneumonia, withdrew at the end of study. Thus, the results of 16 cases and 47 controls (16 healthy subjects, 15 subjects with acute lobar pneumonia, and 16 subjects with asthma) were ultimately analyzed.

The ethnicity of all subjects was the same (Korean). The median age of the children in the cases group (43.8% male) was 26 months (18.25–36.25 months; min, 16 months; max, 60 months). Due to the matched study design, the cases did not differ significantly from the matched control groups in terms of age and sex distribution. None of the subjects had a familial history of idiopathic interstitial pneumonia within the previous 6 months (Table 1). Other socio-demographic and clinical characteristics are described in Table 1. Overall, the cases did not differ significantly from the controls except in terms of day-care center attendance (*P* = 0.04) and a family history of asthma (*P* = 0.01).

**Table 1:** Socio-demographic and clinical characteristics of the 16 children with children’s interstitial lung diseases and their 47 matched controls.

Variables^a^	Cases (n = 16)	Healthy controls (n = 16)	Acute lobar pneumonia (n = 15)	Asthma(n = 16)	*P*
Age (mo), median (IQR)	26.0 (18.0)	26.0 (20.5)	27.0 (18.0)	29.5 (21.0)	0.67
Male gender, n (%)	7 (43.8)	7 (43.8)	6 (40.0)	7 (43.8)	1.00
Educational status of parents, n (%)					0.13
< High school	0	0	0	0	
High school graduate	6 (37.5)	2 (12.5)	3 (20.0)	2 (12.5)	
Some college	4 (25.0)	1 (6.3)	4 (26.7)	6 (37.5)	
College graduate	6 (37.5)	13 (81.3)	8 (53.3)	8 (50.0)	
C/sec delivery, n (%)	4 (25.0)	6 (37.5)	7 (46.7)	10 (62.5)	0.14
Gestational age, n (%)					0.16
>40wks	8 (50.0)	1 (6.3)	4 (26.7)	9 (56.3)	
37–40wks	8 (50.0)	11 (68.8)	9 (60.0)	6 (37.5)	
<37wks	0 (0.0)	4 (25.0)	2 (13.3)	1 (6.3)	
Overseas trip, n (%)	1 (6.3)	2 (12.5)	4 (26.7)	2 (12.5)	0.43
Passive smoking, n (%)					0.44
Current-passive	3 (18.8)	6 (37.5)	1 (6.7)	9 (56.3)	
Ex-passive	4 (25.0)	2 (12.5)	10 (66.7)	1 (6.3)	
No	9 (56.3)	8 (50.0)	4 (26.7)	6 (37.5)	
Day-care center, n (%)	6 (37.5)	9 (56.3)	10 (66.7)	13 (81.3)	0.04*
Pets indoors, n (%)	1 (6.3)	0 (0.0)	0 (0.0)	0 (0.0)	0.25
Oriental medicine, n (%)	3 (18.8)	5 (31.3)	2 (13.3)	6 (37.5)	0.74
Health supplements, n (%)	9 (56.3)	8 (50.0)	3 (20.0)	14 (87.5)	1.00
Vaccination, n (%)					0.09
Yes	14 (87.5)	15 (93.8)	11 (73.3)	14 (87.5)	
No	0 (0.0)	1 (6.3)	3 (20.0)	2 (12.5)	
Unknown	2 (12.5)	0 (0.0)	1 (6.7)	0 (0.0)	
Chronic diseases^b^, n (%)	1 (6.3)	0 (0.0)	1 (6.7)	1 (6.3)	0.79
Factors predisposing to infections^c^, n (%)					
Immune-suppressive therapy	0 (0.0)	0 (0.0)	1 (6.7)	2 (12.5)	0.56
Transplantation	0 (0.0)	0 (0.0)	0 (0.0)	0 (0.0)	
General anesthesia	1 (6.3)	0 (0.0)	0 (0.0)	1 (6.3)	0.45
Transfusion	0 (0.0)	0 (0.0)	0 (0.0)	0 (0.0)	
Antibiotics use in the previous 1 month	13 (81.3)	12 (75.0)	8 (53.3)	13 (81.3)	0.53
Admission	3 (18.8)	1 (6.3)	1 (6.7)	2 (12.5)	0.36
Family history, n (%)					
ILD^d^	0 (0.0)	0 (0.0)	0 (0.0)	0 (0.0)	1.00
Asthma	0 (0.0)	5 (31.3)	2 (13.3)	9 (56.3)	0.01
Other allergic diseases	8 (50.0)	12 (75.0)	8 (53.3)	16 (100.0)	0.06
Chronic respiratory diseases in the previous 1 year^e^	0 (0.0)	0 (0.0)	1 (6.7)	0 (0.0)	0.65

a Variables in the previous 1 year.

b The occurrence of any of the following in the previous 6 months: autoimmune disease, chronic liver disease, chronic renal insufficiency, neurologic diseases with immobility, congenital heart disease, chronic lung disease, diabetes mellitus or malignancy.

c Factors predisposing to infections in the previous 6 months.

d Interstitial lung disease in the 6 months prior to or after index date.

e Bronchiectasis, chronic obstructive pulmonary disease, chronic bronchitis, pulmonary tuberculosis, pertussis or cystic fibrosis.

* *P*<0.05.

Indoor/outdoor environmental factors as risk factors for chILD.

The cases and controls did not differ significantly with respect to indoor environmental factors, namely, housing (*P* = 0.64), storey of residence (*P* = 0.75), age of the residence (*P* = 0.66), number of rooms (*P* = 0.77), or presence of visible mold indoors (*P* = 0.58). Cases and controls also did not differ with respect to prominent sources of chemical compounds (heating system: *P* = 0.99, house renovation: *P* = 0.64, new furniture: *P* = 0.42, air cleanser: *P* = 0.22, air conditioner: *P* = 0.54, air conditioner mold cleanser: *P* = 0.37, water purifier: *P* = 0.81, and mosquitocide: *P* = 0.23), but did differ for exposure to use of hair spray (*P* = 0.00). Significant differences were observed with respect to the use of humidifiers and HDs in the 12 months before the index date. In fact, a humidifier was used by the families of all chILD before the index date; by contrast, only 75.0% of the healthy controls, 73.3% of acute lobar pneumonia, and 56.3% of asthma used humidifiers (*P* = 0.02). Moreover, the chILD families reported that all had used HDs in the 12 months before the index date; this frequency was significantly higher than that of the controls (*P* = 0.00) (Table 2). By contrast, outdoor environmental factors, including living region and outside facilities within 2 km, were not significantly different among the study subjects (Table 3).

**Table 2:** Comparison of indoor environmental factors between the16 children with children’s interstitial lung diseases and their 47 matched control.

Variables^a^	Cases (n = 16)	Healthy controls (n = 16)	Acute lobar pneumonia (n = 15)	Asthma (n = 16)	*P*
Housing, n (%)					0.64
Town house	0 (0.0)	1 (6.3)	0 (0.0)	1 (6.3)	
Apartment	14 (87.5)	12 (75.0)	10 (66.7)	13 (81.3)	
Multiplex house	1 (6.3)	3 (18.8)	4 (26.7)	2 (12.5)	
Mixed-use apartment	1 (6.3)	0 (0.0)	1 (6.7)	0 (0.0)	
Living storey, n (%)					0.75
Underground/semi-underground	0 (0.0)	0 (0.0)	0 (0.0)	0 (0.0)	
1st to 3rd floor	3 (18.8)	5 (31.3)	5 (33.3)	7 (43.8)	
4th to 6th floor	4 (25.0)	5 (31.3)	3 (20.0)	2 (12.5)	
7th floor and higher	9 (56.3)	6 (37.5)	7 (46.7)	7 (43.8)	
Heating system, n (%)					0.99
Central heating	4 (25.0)	4 (25.0)	4 (26.7)	4 (25.0)	
Local heating, gas	12 (75.0)	11 (68.8)	10 (66.7)	12 (75.0)	
Local heating, oil	0 (0.0)	1 (6.3)	1 (6.7)	0 (0.0)	
Local heating, coal	0 (0.0)	0 (0.0)	0 (0.0)	0 (0.0)	
Drinking water, n (%)					
Tap water	7 (43.8)	4 (25.0)	7 (46.7)	9 (56.3)	0.34
Well water	0 (0.0)	0 (0.0)	0 (0.0)	0 (0.0)	1.00
Groundwater	0 (0.0)	0 (0.0)	1 (6.7)	0 (0.0)	0.24
Bottled-mineral water	5 (31.3)	6 (37.5)	0 (0.0)	2 (12.5)	0.03*
Home-purified water	6 (37.5)	8 (50.0)	8 (53.3)	8 (50.0)	0.82
Age of house, n (%)					0.66
<1 year	1 (6.3)	0 (0.0)	0 (0.0)	0 (0.0)	
2–5 years	2 (12.5)	4 (25.0)	5 (33.3)	2 (12.5)	
6–10 years	6 (37.5)	3 (18.8)	3 (20.0)	5 (31.3)	
>10 years	6 (37.5)	9 (56.3)	7 (46.7)	9 (56.3)	
Unknown	1 (6.3)	0 (0.0)	0 (0.0)	0 (0.0)	
Numbers of rooms, n (%)					0.77
1	0 (0.0)	0 (0.0)	0 (0.0)	0 (0.0)	
2	5 (31.3)	4 (25.0)	4 (26.7)	3 (18.8)	
3	10 (62.5)	9 (56.3(	10 (66.7)	12 (75.0)	
>3	1 (6.3)	3 (18.8)	1 (6.7)	1 (6.3)	
House renovation, n (%)	0 (0.0)	2 (12.5)	1 (6.7)	2 (12.5)	0.64
New furniture, n (%)	5 (31.3)	6 (37.5)	8 (53.3)	9 (56.3)	0.42
Indoor mold, n (%)	3 (18.8)	5 (31.3)	6 (40.0)	6 (37.5)	0.58
Carpet in the house, n (%)	0 (0.0)	2 (12.5)	2 (13.3)	4 (25.0)	0.22
Air cleaner, n (%)	6 (37.5)	7 (43.8)	3 (20.0)	9 (56.3)	0.22
Air conditioner, n (%)	11 (68.8)	14 (87.5)	12 (80.0)	11 (68.8)	0.54
Air conditioner mold cleanser, n (%)	3 (27.3)	3 (21.4)	2 (16.7)	0 (0.0)	0.37
Water purifier, n (%)	7 (43.8)	8 (50.0)	9 (60.0)	9 (56.3)	0.81
Humidifier, n (%)	16 (100.0)	12 (75.0)	11 (73.3)	9 (56.3)	0.02*
Type of humidifier, n (%)					0.32
Ultrasonic	7 (43.8)	3 (18.8)	2 (13.3)	4 (25.0)	
Complex	5 (31.3)	2 (12.5)	4 (26.7)	2 (12.5)	
Steam	0 (0.0)	3 (18.8)	1 (6.7)	3 (18.8)	
Natural	0 (0.0)	2 (12.5)	2 (13.3)	0 (0.0)	
Unknown	4 (25.0)	6 (37.5)	6 (40.0)	7 (43.8)	
Humidifier disinfectant, n (%)	16 (100.0)	3 (18.8)	3 (20.0)	5 (31.3)	0.00*
Air freshener, n (%)	6 (37.5)	8 (50.0)	5 (33.3)	5 (31.3)	0.70
Hair spray, n (%)	2 (12.5)	3 (18.8)	9 (60.0)	0 (0.0)	0.00*
Aromatic candle, n (%)	0 (0.0)	3 (18.8)	2 (13.3)	4 (25.0)	0.20
Mosquitocide, n (%)	9 (56.3)	9 (56.3)	12 (80.0)	13 (81.3)	0.23
Type of mosquitocide, n (%)					
Coil	1 (6.3)	0 (0.0)	0 (0.0)	1 (6.3)	1.00
Electronic mat	5 (31.3)	4 (25.0)	7 (46.7)	4 (25.0)	0.55
Electronic liquid	4 (25.0)	6 (37.5)	3 (20.0)	7 (43.8)	0.49
Spray	0 (0.0)	1 (6.3)	3 (20.0)	2 (12.5)	0.17
Herbicide, n (%)	0 (0.0)	0 (0.0)	0 (0.0)	0 (0.0)	1.00

a Variables in the previous 1 year.

* *P*<0.05.

**Table 3:** Comparison of outdoor environmental factors between the16 children with children’s interstitial lung diseases and their 47 matched controls.

Variables^a^	Cases (n = 16)	Healthy controls (n = 16)	Acute lobar pneumonia (n = 15)	Asthma (n = 16)	*P*
Living region, n (%)					0.90
Urban area, residential district	14 (87.5)	15 (93.8)	15 (100.0)	14 (87.5)	
Urban area, business district	1 (6.3)	1 (6.3)	0 (0.0)	2 (12.5)	
Rural area	1 (6.3)	0 (0.0)	0 (0.0)	0 (0.0)	
Industrial area	0 (0.0)	0 (0.0)	0 (0.0)	0 (0.0)	
Outside facilities within 2 km, n (%)	3 (25.0)	3 (25.0)	3 (25.0)	3 (25.0)	1.00
Garbage incinerator	0	1 (6.3)	0	0	1.00
Sewage treatment plants	1 (6.3)	0	1 (6.7)	0	0.61
Factory	1 (6.3)	1 (6.3)	0	1 (6.3)	1.00
Chemical treatment plants	0	0	1 (6.7)	0	0.24
Farm/orchard	2 (12.5)	1 (6.3)	0	2 (12.5)	0.74
Cattle shed/pigsty	0	1 (6.3)	0	0	1.00
Power station	0	1 (6.3)	0	0	1.00

a Variables in the previous 1 year.

Univariate analysis of the association between the use of HDs and the risk of chILD.

In conditional regression models, none of the socio-demographic, personal, or indoor/outdoor variables were associated with risk of chILD but the risk associated with the previous use of HDs was significant (OR, 2.73; 1.41–5.90, *P* = 0.00) (Table 4). Attendance in day-care center and previous use of hair spray were not associated with risk of chILD in the conditional regression model (0.69; 0.36–1.30, *P* = 0.25, 0.83; 0.43–1.61, *P* = 0.59).

**Table 4:** Univariate analysis of the association between the use of a humidifier disinfectant and the risk of children’s interstitial lung diseases in 1∶3 matched case and control.

Variables^a^	Cases (n = 16)	Controls (n = 47)	Unadjusted OR^b^ (95% CI)	*P*
Humidifier disinfectant, n (%)	16 (100.0)	11 (34.4)	2.73 (1.41–5.90)	0.00
Humidifier, n (%)	16 (100.0)	32 (68.1)	1.46 (0.77–2.76)	0.25
Oriental medicine	3 (18.8)	13 (27.7)	0.87 (0.42–1.82)	0.71
Passive smoking, n (%)	7 (43.8)	29 (61.7)	0.80 (0.42–1.51)	0.49
Health supplement, n (%)	9 (56.3)	25 (53.2)	1.03 (0.59–1.80)	0.91
Day-care center, n (%)	6 (37.5)	32 (68.1)	0.69 (0.36–1.30)	0.25
Factors predisposing to infections c, n (%)	14 (81.3)	35 (74.5)	1.13 (0.55–2.35)	0.74
Chronic diseases d, n (%)	1 (6.3)	2 (4.3)	1.14 (0.28–4.57)	0.86
Heating system, n (%)				
Central heating	4 (25.0)	12 (25.5)	1.0 (referent)	
Local heating, gas	12 (75.0)	33 (70.2)	1.01 (0.52–1.96)	0.98
Local heating, oil	0 (0.0)	2 (4.3)	0.74 (0.13–4.19)	0.73
Drinking water, n (%)				
Tap water	5 (31.3)	14 (29.8)	1.0 (referent)	
Ground water	0 (0.0)	1 (2.1)	0.70 (0.07–6.97)	0.76
Bottled-mineral water	5 (31.3)	8 (17.0)	1.22 (0.53–2.80)	0.65
Home-purified water	6 (37.5)	24 (51.1)	0.93 (0.49–1.76)	0.82
New furniture in the previous 1 year, n (%)	5 (31.3)	23 (48.9)	0.85 (0.49–1.48)	0.56
Indoor mold in the previous 1year, n (%)	3 (18.8)	17 (36.2)	0.79 (0.40–1.55)	0.49
Water purifier, n (%)	7 (43.8)	26 (55.3)	0.89 (0.50–1.57)	0.68
Air cleaner, n (%)	6 (37.5)	19 (40.4)	0.97 (0.54–1.72)	0.90
Air freshener, n (%)	6 (37.5)	18 (38.3)	0.99 (0.49–2.00)	0.98
Air conditioner, n (%)	11 (68.8)	37 (78.7)	0.84 (0.41–1.73)	0.64
Air conditioner mold cleanser, n (%)	3 (18.8)	5 (10.6)	1.20 (0.51–2.84)	0.68
Hair spray, n (%)	2 (12.5)	12 (25.5)	0.83 (0.43–1.61)	0.59
Mosquitocide, n (%)	9 (56.3)	34 (72.3)	0.80 (0.40–1.59)	0.52
Outside facilities^e^, n (%)	3 (18.8)	9 (19.2)	0.99 (0.45–2.16)	0.97

a Variables in the previous 1 year.

b Univariate analysis; conditional logistic regression analysis.

c Immune-suppressive therapy, general anaesthesia, transfusion, admission within 6 months, and prior use of antibiotics within 1 month.

d The occurrence of any of the following in the previous 6 months: autoimmune disease, chronic liver disease, chronic renal insufficiency, neurologic diseases with immobility, congenital heart disease, chronic lung disease, diabetes.

e Garbage incinerator, sewage treatment plants, factory, chemical treatment area, plants, farm/orchard, cattle shed/pigsty, and power station.

## Discussion

In the present study, HD use, but not humidifier use, was independently associated with an increased risk of chILD. Other indoor/outdoor environmental factors, including other sources of chemicals, did not show similar associations. To our knowledge, this is the first case-control study that has investigated the relationship between HD use and the risk of chILD.

Humidifiers are commonly used at home and in offices to relieve respiratory symptoms and skin dryness, particularly in young children and pregnant women. The MOCEH birth cohort in Korea reported that 28.2% of the pregnant women surveyed used humidifiers, and a rate that increased to over 45% in winter. [7] This wide-spread use of humidifiers has raised concerns about associated lung diseases, such as complicated bacterial or fungal pneumonia. [8].

In Korea, HDs were originally introduced as industrial products but they were quickly adopted for private use to prevent or suppress the growth of molds, bacteria, or algae in water tanks, albeit without warnings or risk assessments. [9] A cross-sectional study conducted in Korea reported that of the humidifier users, 18.1% also used HDs, and that this frequency increased to over 30% in winter. [10].

HDs contain oligo [2-(2-ethoxy) ethoxyethyl] guanidium chloride (PGH), polyhexamethyleneguanidine (PHMG) and didecyldimethylammonium chloride (DDAC). [9], [11] Their manufacturers recommend their dilution, typically 5 mL of HD in 3 L of distilled water. PHMG and PGH are well-known disinfectants for the treatment of various medical conditions, and their use dates back to the late 19th century. They have been classified as nontoxic crystalline compounds for oral intake or skin application. [12] Polyhexamethylene biguanidine (PBMB), a polymer of PHMG, is a broad-spectrum antimicrobial agent that induces membrane damage and genomic alterations in microorganisms. [13] It is very stable in water, even after 60 days, [14] and is a common ingredient in various consumer products (e.g., shampoo, skin antiseptics, and sanitizers of water systems and swimming pools) due to its reported safety by the oral or skin routes of exposure. [14] It has also been investigated as a stabilizing ligand for the synthesis of silver nanoparticles (Ag NPs). [15] There is only scant information on the toxicity of PHMG. The US Environmental Protection Agency (EPA) has never approved the use of PHMG in diluted form to prevent humidifier-associated lung disease. Rather, the agency has reported that the acute and chronic dietary risks of PBMB are below the level of concern. [16] However, there are reports that PHMG, as an alcohol surrogate, induces disorders of lipid metabolism and may lead to permanent, even fatal, liver injury, as was the case in several Russians who consumed illegally produced alcohol. [17] In addition, the acute cardiovascular toxicity of HDs in Zebrafish was reported recently: in this model, exposure to PHMG and PGH, even at normal doses, causes severe atherosclerotic changes, has a cytotoxic effect on human dermal cells, and induces severe vascular fibrosis, inflammation, and embryonic toxicity. [18] This report may support our findings that suggesting pulmonary fibrosis was caused by inhalation toxicity of HDs.

DDAC, as a broad-spectrum microbial agent, is used in many applications (e.g. water systems and swimming pools, medical and surgical sterilization, humidifiers, and commercial aerosol sprays). [19] The toxicity of DDAC has been well evaluated and seems to be dose dependent. Animal studies have demonstrated that oral intake causes skeletal alterations, Moreover, humans and animals can develop contact dermatitis after topical use. The EPA has reported that inhalation toxicity, although still not fully understood, appears to be dose dependent. [19] Recently, acute pulmonary injury was induced in a mouse model after the dose- and time-dependent administration of DDAC. [20] In that study, when DDAC was administered by the intratracheal route at a sublethal dose (150 µg/kg), it exhibited cytotoxic, proinflammatory, and profibrogenic properties, and was lethal to alveolar macrophages and parenchymal cells.

During their lifetime, humans are exposed to tens of thousands of synthetic chemicals, many of them recently developed, which are used in countless applications. While the benefit or risk to human health of some chemicals has either been proven or an association has been established, in most cases, their effects on human health have been scarcely evaluated and thus are only poorly understood. [21] This has raised concern; given that children have shown parallel increases in the incidences of asthma, neuro-developmental diseases, preterm birth, and birth defects. [22].

The EPA has identified 3,000 high-production-volume (HPV) chemicals [23] and the European Union has established the Registration, Evaluation, Authorization and Restriction of Chemical substances (REACH) to protect human health and to evaluate the hazards that are posed by synthetic chemicals. [24] However, these risk assessment and the determination of toxic concentrations are mostly defined for adults, despite the fact that children are clearly exposed to toxic chemicals in the environment at higher levels because of their inherent susceptibility (i.e. larger body surface area in proportion to their weight, the critical window for their development and growth, and their physiological immaturity). [25], [26].

In the “Magic Nano” issue reported in Germany in 2006, it was asserted that co-evaporation of nanoparticles with active components and solvents could cause pulmonary toxicity in humans [27] and animals. [28] Furthermore, a recent report found that the “white dust” generated by humidifiers can cause lung injury in young infants. [29] The small size of nanoparticles (≤100 nm in diameter) makes the respiratory system particularly vulnerable to nanoparticle deposition. Although nanotechnology has been applied to diverse fields, such as medicine and engineering, with consequent increases in human prosperity and well-being, the rapid advancement of nanotechnology is certain to raise new safety concerns, especially with regard to the respiratory system. [30] Crucially, in 2011, the KCDC reported that 30–80 nm nanoparticles consisting of PHMG or PGH are generated when HDs are added to the distilled water used in ultrasonic humidifiers. [31] These aerosolized HDs probably decompose into nano-sized particles that are subsequently deposited in the respiratory system, where they may cause chILD.

As a retrospective study, our study has inherent limitations: 1) The subjects and controls do not represent the general population, 2) The retrospective, medical record-based ascertainment of cases and controls may be subject to its own limitations; 3) The parents/guardians of the cases and the controls were not completely blinded to the purpose of this study when they answered the questionnaire because this study was designed after May 11, 2011, when the serial media reports of HDs and their relationship with highly fatal ILD were released and brought the matter to the attention of the entire nation. Moreover, the interviewing process started after the KCDC announcement on August 31 2011 that HDs may be the cause of ILD in adults. However, while the media reports and the announcement may have raised the interest of the caregivers of cases, which could have led to some bias (e.g. recall or information bias), it is likely that the announcement had equivalent effects on the caregivers of the controls. Nevertheless, to reduce this possibility, patients with acute lobar pneumonia and asthma were selected as non-healthy controls. The three controls did not differ significantly in terms of use of HDs (18.8% in the healthy controls, 20.0% in the acute lobar pneumonia group, and 31.3% in the asthma group, *P* = 0.75 in table 2); 4) Finally, there was no adjustment for unmeasured confounding or potential variables, such as environmental factors in day-care centers, outdoor air quality, and fine dust particles in the homes. However, becuase a prospective observational or clinical interventional study is impossible due to ethical issues and the low incidence of chILD, concerted efforts were made to minimize any expected selection or information biases. A case was confirmed only when the members of the expert committee unanimously agreed on the diagnosis, while controls in the three groups were randomly selected from the computerized database to reduce selection bias and to increase comparability. The disease in both the cases and the non-healthy controls occurred recently (2010–2011). Control children with acute lobar pneumonia or asthma who had been admitted to our institutions at the index date, were selected to avoid a lack of information. To minimize recall bias, an illustrated questionnaire was developed, including photographs of all indoor/outdoor variables.

The design of the present study did not allow us to determine the extent to which the risk of chILD depended on the amount of HD exposure, nor could we speculate about an interaction between a genetic predisposition and HDs. However, because the cases were clustered in spring, likely concentrated use of HDs in winter, and only a few of the numerous HDs users were affected by chILD, it seems likely that either exposure dose or a genetic predisposition plays an important role in the pathogenesis of this disease. Although our study population is not necessarily representative of the general population in Korea, the results point to a strong association between HDs and the development of chILD. Fortunately, there have been no further cases of chILD in Korea since January 1, 2012, when the KCDC banned the sale of HDs beginning in November, 2012 (data not shown). The continued absence of such cases will provide indirect supporting evidence for an association between HD use and chILD in Korea.

Taken together, our findings indicate that the use of HDs in winter is strongly associated with an increased risk of chILD. Little is known about the potential toxicity of HDs and other chemicals when exposure occurs through an unexpected route and the synergistic adverse effects due to combined exposures remain unclear. The present study suggests that environmental chemicals that may be potentially hazardous to human health should be examined more closely.

In conclusion, we provide additional evidence that environmental chemicals may pose an unanticipated hazard to human health if their use in specific applications is not closely monitored. Therefore, continuous monitoring and, if needed, changes in policy are essential to prevent and control pediatric diseases caused by toxic chemicals.

## Supporting Information

Material S1 **Conceptual framework and study process.**
(DOCX)

Material S2 **Questionnaire evaluating the effects of exposure to various environmental factors on human health.**
(DOCX)
