# Supplementary material for: Inhalation Toxicity of Humidifier Disinfectants as a Risk Factor of Children’s Interstitial Lung Disease in Korea: A Case-Control Study
Source: PLoS One. 2013 Jun 5;8(6):e64430. doi: 10.1371/journal.pone.0064430 (PMC3673956; doi:10.1371/journal.pone.0064430)
Supplement: Material S1 — Conceptual framework and study process. (DOCX) [file pone.0064430.s001.docx]

**Supplementary materials**

*Conceptual framework*

Sporadic epidemics of a unique type of chILD with unknown causes have been observed in spring in South Korea since 2006. Several studies have been performed to characterize and identify the cause of this disease.[[1](#_ENREF_1),[2](#_ENREF_2)]

In spring 2006, an epidemic of acute interstitial pneumonia in children that was characterized by similar clinico-radiologic-pathological findings[such as rapidly progressing pulmonary fibrosis and alveolar damage, air leakage and high mortality (47%)] was observed in South Korea.[[1](#_ENREF_1)] The clinico-radiologic-pathological findings of this form of chILD were unique and were not consistent with any other diagnosis. Unfortunately, epidemics of this form of chILD recurred every spring after 2006.Therefore, in 2008, prospective nationwide surveillance was performed to identify the cause and proper treatment of chILD. This surveillance revealed 78 retrospective cases and nine new cases.[[2](#_ENREF_2)]Despite concentrated efforts, the cause could not be identified. However, analysis of the cases revealed that the natural course of chILD was characterized by rapid progression and high mortality. Then, in late April 2011, a dozen young pregnant and postpartum women in South Korea developed ILD. Despite full work-up, potentially causative microorganisms and/or underlying conditions could not be identified. Crucially, the cases identified by the surveillance included five pairs of postpartum women and their children who were both affected with a form of ILD that was similar in terms of clinico-radiologic-pathological features.[[3](#_ENREF_3)]For this reason, it was hypothesized that the risk factors of chILD in South Korea were likely to be one or more indoor environmental factors to which household members are evenly exposed.

*Study design*

The present study was conducted as a case-control study because of the rare incidence of the disease. Each case was matched with three control subjects, one each from three parallel control groups that differed in terms of disease category: they were composed of healthy subjects, patients with acute lobar pneumonia, and patients with asthma. These controls were selected to reduce recall/information bias. The cases and controls were matched according to age, gender, and index date to reduce potential confounders such as season, duration between exposure and disease, and other unmeasured variables (e.g., yellow dust in spring).

*Development of the questionnaire*

A questionnaire was developed by a committee that consisted of pediatric pulmonologists and epidemiologists. The questionnaire consisted of two parts, namely, questions on socio-demographic/personal information, and questions on indoor/outdoor environmental factors. Since the questionnaire was developed before the KCDC report on August 31, 2011, questions about various exposure variables to which household members could be evenly exposed were included. These variables included the use of air conditioners, air cleaners, water purifiers, humidifiers, and disinfectants used in these home appliances.

*Study process*

This study was designed before the public became aware of the potential hazard of HDs. However, the interviewing process started after the KCDC announced that HDs may be the cause of ILD in adults. Thus, although it was originally intended that this study should evaluate the role HD use plays in chILD pathogenesis by blinded interviews, blinding in the present study was not successful because of the public awareness of and national attention to this issue.

*Acquisition of questionnaire data*

The interviews with the parents or caregivers were conducted by three trained interviewers. They did not respond to the caregivers’ questions and only provided support to help the interviewees remember the index date and the exposure to the variables before the index date.

*References*

1. Cheon CK, Jin HS, Kang EK, Kim HB, Kim BJ, et al. (2008) Epidemic acute interstitial pneumonia in children occurred during the early 2006s. *Korean J Pediatr* 51: 383-390.

2. Kim BJ, Kim HA, Song YH, Yu J, Kim S, et al. (2009) Nationwide surveillance of acute interstitial pneumonia in Korea. *Korean J Pediatr* 52: 324-329.

3. Lee E, Seo J, Kim H, Yu J, Song J, et al. (2012) Two series of familial cases with unclassified interstitial pneumonia with fibrosis. *Allergy Asthma Immunol Res* 4: 240-244.
