# Supplementary material for: Inhalation Toxicity of Humidifier Disinfectants as a Risk Factor of Children’s Interstitial Lung Disease in Korea: A Case-Control Study
Source: PLoS One. 2013 Jun 5;8(6):e64430. doi: 10.1371/journal.pone.0064430 (PMC3673956; doi:10.1371/journal.pone.0064430)
Supplement: Material S2 — Questionnaire evaluating the effects of exposure to various environmental factors on human health. (DOCX) [file pone.0064430.s002.docx]

**FORM 2: Indoor and outdoor environmental factors**

**Your child’s index date is ______/_______/_______**

**This questionnaire was developed to evaluate the effects of exposure to various environmental factors on human health**

**Please answer every question**

**Please only describe the exposure that occurred BEFORE the index date**

**Your child’s index date is ______/_______/_______**

**Author: the text “In exposures after index date, would you answer NO” is a bit unclear. Do you mean: “Please only describe the exposure that occurred BEFORE the index date”?**

**________________________:(NAME_LAST) LAST NAME**

**________________________:(NAME_FIRST) FIRST NAME**

**________________________:(NAME_INTERVIEWER) FULL NAME**

**_______/_______/_________:(DATE_INTERVIEWER) mm/dd/yyyy**

**Information on residence**

1) Where did your child (he/she) live?

① Urban area, residential district ② Urban area, business district

③ Rural area ④ Industrial area

2)What was the linear distance between the home and the nearest main street (four lanes or more)?

① Less than 10 m ② 11–50 m

③ 51–100 m ④ 101–200 m

⑤ 201–500 m ⑥ More than 501 m

⑦ Do not know

3) Which facilities were within 2 km from the home? (Please select all applicable answers.)

① Garbage incinerator ② Sewage treatment plants

③ Factory ④ Chemical substance treatment plants

⑤ Farm/orchard ⑥ Cattle shed/pigsty

⑦ Power station ⑧ Do not know

4) What kind of building did your child live in?

①Town house ② Apartment

③ Multiplex house ④ Mixed-use apartment

5) On which floor did he (she) live?

① Basement/semi-basement ② 1^st^–3^rd^ floor

③ 4^th^–6^th^ floor ④ 7^th^ floor and above

6) How many rooms were there in the home?

① 1 room ② 2 rooms

③ 3 rooms ④ 4 or more rooms

7) How old was the building your child was living in?

① Under 1 year ② 2–5 years

③ 6–10 years ④ Over 10 years

⑤ Do not know

8) What kind of heating system was installed?

① Central heating ② Local heating, gas boiler

③ Local heating, oil boiler ④ Local heating, briquette

⑤ Do not know

9) Where was the kitchen located?

① Indoors, separate from the living room ② Indoors, combined with the living room

③ Outdoors (e.g.,veranda)

10) What kinds of water did your child drink?

① Tap water ② Well water

③ Groundwater ④ Bottled-mineral water

⑤ Home-purified water ⑥ Do not know

11) Did your child drink boiled water in the home?

① Yes ② No

**Information on the indoor environment**

12) Had you renovated the home in which your child was living in the previous 12 months?

① Yes ② No (☞go to question 13)

12-1) If yes, when did you renovate?

① Less than 6 months ago ② 6 to 12 months ago

13) Had you bought new furniture and placed it in the room in which your child was living in the previous 12 months?

① Yes ② No (☞go to question 14)

13-1) If yes, when did you buy the new furniture?

① Less than 6 months ago ② 6 to 12 months ago

14) Had you changed the wallpaper in the home in the previous 12 months?

① Yes ② No (☞go to question 15)

14-1) If yes, when did you change the wallpaper?

① Less than 6 months ago ② 6 to 12 months ago

15) Were there wet stains or mold in any of the rooms that were used by your child in the previous 12 months??

① Yes ② No (☞go to question 16)

15-1) If yes, please tick a box in the following table to indicate where and how much.

(If there were no such stains or the room(s) with stains was not used by your child, please tick the 0% box)

| Where/how much | Not visible (0%) | 5% or less | 5–30% | 30% or more |
| --- | --- | --- | --- | --- |
| Sleeping/living room | ① | ② | ③ | ④ |
| Kitchen | ① | ② | ③ | ④ |
| Bathroom | ① | ② | ③ | ④ |
| Others | ① | ② | ③ | ④ |

Others: utility room, laundry, veranda, basement, etc.

15-2) Had there been a smell of mold at the home in the previous 12 months?

① Never ② Sporadically ③ Mildly ④ Intensely

15-3) Had there been water leakage or wet spots in the home in the previous 12 months?

① Yes ② No

16) Had there been carpets in the home in the previous 12 months?

① Yes ② No (☞go to question 17)

16-1) If yes, where are/were the carpets?

① Living room ② Adult’s room ③ Child’s room ④ Entrance

⑤ Others

If ⑤, describe ______________________

17) Had you used an air cleaner at the home in the previous12 months?

① Yes ② No (☞go to question 18)

17-1)If yes, where did you use it?

① Living room ② Adult’s room ③ Child’s room ④Others

If ④, describe ______________________

17-2) How many hours a day did you use it?________________hours/day (describe)

17-3) How many days a week did you use it?________________days/week (describe)

18) Had you used an air conditioner at the home in the previous 12 months?

① Yes ② No (☞go to question 19)

18-1) What kind of air conditioner did you use?

① System type ② Stand type ③ Window type

④ Portable type ⑤ Others ⑥ Do not know

If ⑤, describe ______________________

18-2) How many hours a day did you use it?________________hours/day (describe)

18-3) How many days a week did you use it?________________days/week (describe)

18-4) Had you used an air conditioner fungicide?

① Yes ② No

19) Had you used a water purifier at the home in the previous12 months?

① Yes ② No (☞go to question 20)

19-1)If yes, did your child drink the water from the purifier unboiled or boiled?

① Unboiled ② Boiled

19-2) Which of the following brands of water purifier were used in the home?


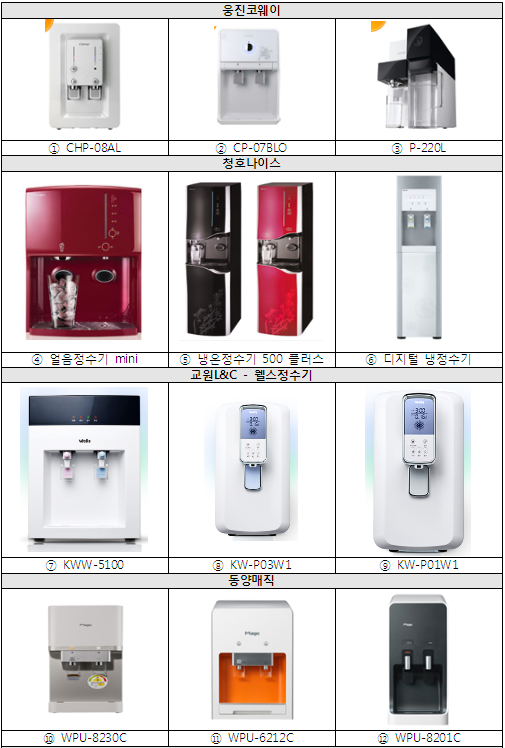


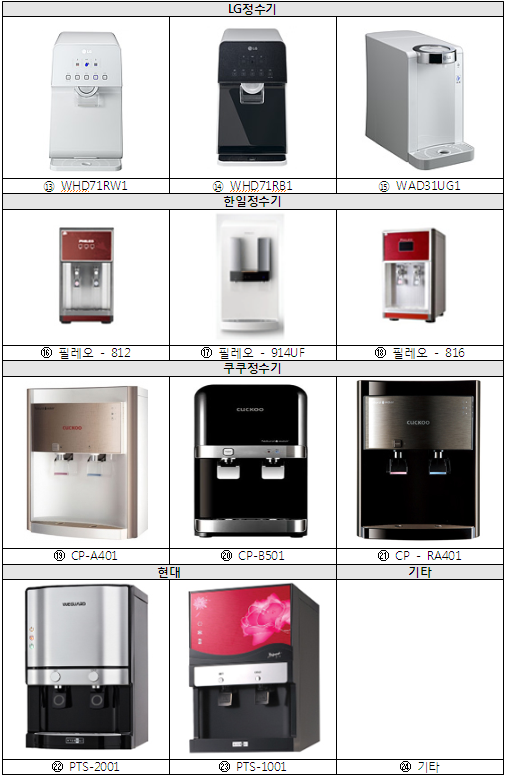


20) Had you used a humidifier at the home in the previous 12 months?

① Yes ② No (☞go to question 21)

20-1) What kind of the humidifier was used in the home?

① Ultrasonic type ② Steam type ③ Complex type

④ Evaporative type ⑤ Others ⑥ Do not know

If ⑤, describe ______________________

20-2) What kind of water did you use for the humidifier?

① Unboiled tap water ② Boiled tap water

③ Unboiled purified water ④ Boiled purified water

⑤ Bottled mineral water ⑥ Do not know

20-3) Where was the humidifier placed? (Please select all applicable answers.)

① Living room ② Adult’s room ③ Child’s room ④ Others

If ④, describe ______________________

20-4) How many hours a day did you use it?________________hours/day (describe)

20-5) How many days a week did you use it?________________days/week (describe)

20-6) How often do/did you clean the humidifier?

① Everyday ② Every other day ③ Every three days

④ Once a week ⑤ Once a month ⑥ Never

21) Had you used commercial humidifier disinfectants for the home humidifier in the previous 12 months?

① Yes ② No (☞go to question 22)

21-1) How many hours a day did you use it?________________hours/day (describe)

21-2) How many days a week did you use it?________________days/week (describe)

21-3) Which of the following brands of humidifier disinfectant were used for the humidifier in the home?


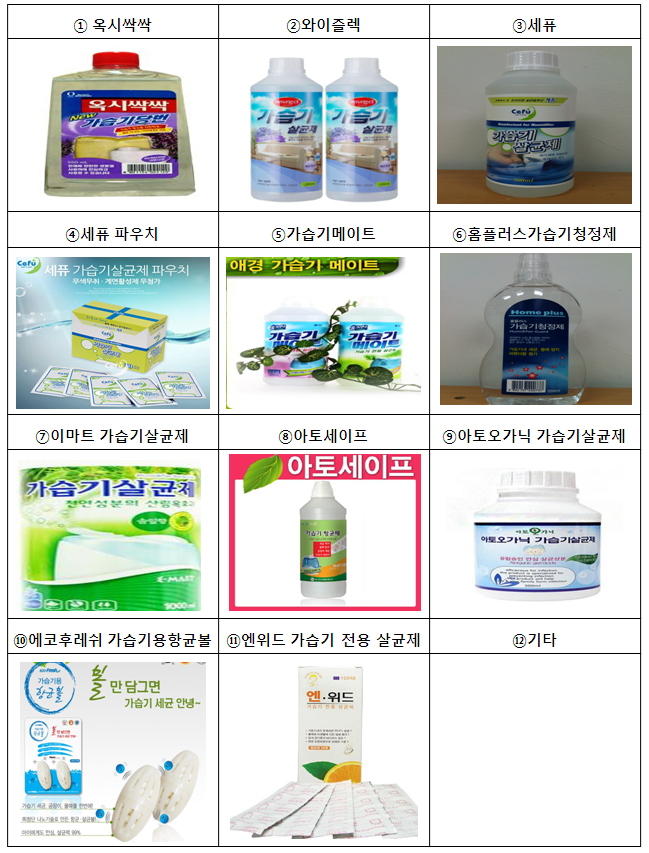


22) Had you used air freshener in the home in the previous12 months?

① Yes ② No (☞go to question 23)

22-1) Where did you use the air freshener? (Please select all applicable answers.)

①Living room ② Adult’s room ③Child’s room ④Others

If ④, describe ______________________

22-2) How many hours a day did you use it?________________hours/day (describe)

22-3) How many days a week did you use it?________________days/week (describe)

22-5) Which of the following brands of air freshener were used in the home?

| ① 한국존슨 크리스탈 로맨스 | ② 페브리즈 비치형 | ③ 산도깨비 향기속으로 |
| --- | --- | --- |
| 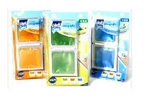 | 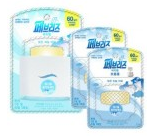 | 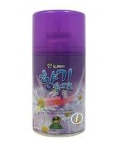 |
| ④ 페브리즈 에어 | ⑤ 페브리즈 조명용 방향제 | ⑥ 옥시 에어윅 아로마 |
| 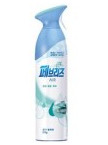 | 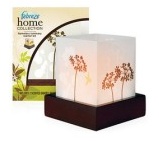 | 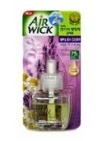 |
| ⑦ 향그린 | ⑧ 옥시 에어윅 펄 방향제 | ⑨ 애경 에어 후레쉬 |
| 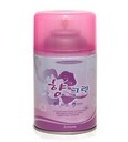 | 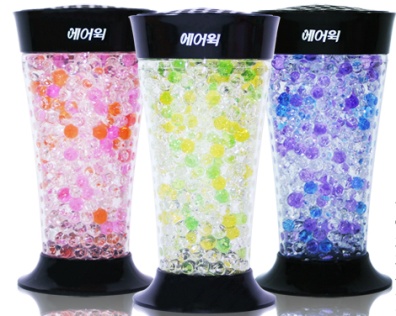 | 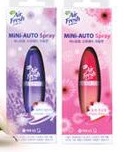 |
| ⑩ 옥시 에어윅 아로마겔 | ⑪ 산도깨비 하우스 에어 | ⑫ 기타 |
| 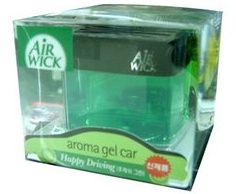 | 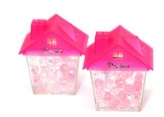 |  |

23) Had you used burning aromatic products such as scented candles or incense in the home in the previous12 months?

① Yes ② No (☞go to question 24)

23-1) Where did you use these aromatic products? (Please select all applicable answers.)

① Living room ② Adult’s room ③ Child’s room ④ Others

If ④, describe ______________________

23-2) How many hours a day did you use it?________________hours/day (describe)

23-3) How many days a week did you use it?________________days/week (describe)

24) Had you used mosquitocides in the home in the previous 12 months?

① Yes ② No (☞go to question 25)

24-1) Where did you use the mosquitocides? (Please select all applicable answers.)

① Living room ② Adult’s room ③ Child’s room ④ Others

If ④, describe ______________________

24-2) How often did you use them?

| (a) In summer | (b) In other seasons |
| --- | --- |
| ①Never | ①Never |
| ②Once or twice a week | ②Once or twice a week |
| ③Three or four times a week | ③Three or four times a week |
| ④More than five times a week | ④Five to seven times a week |

24-3) What type of product did you use? (Please select all applicable answers.)

① Mosquito coil ② Mosquito electrical mat

③ Mosquito liquid ④ Mosquito Spray

25) Had your child used hair styling products in the previous 12 months?

① Yes ② No (☞go to question 26)

26) Had your child been exposed to pesticides/herbicides/household fertilizer in the previous 12 months?

① Yes ② No (☞go to question 27)

26-1) What type of chemical was your child exposed to?

① Pesticide ② Herbicide ③ Household fertilizer

27) Had your child lived with a pet at the home in the previous12 months?

① Yes ② No

27-1) If yes, what kind of animal was it?

① Dog ② Cat ③ Another furry animal ④ Others

If ④, describe ______________________

27-2) Where did you keep the animal?

① Living room ② Adult’s room ③ Child’s room ④ Others

If ④, describe ______________________

27-3) How long have you had the animal?________________months (describe)

We would greatly appreciate your answering the questionnaire.
